# Diarrhea with deficiency kidney-yang syndrome caused by adenine combined with *Folium senna* was associated with gut mucosal microbiota

**DOI:** 10.3389/fmicb.2022.1007609

**Published:** 2022-10-11

**Authors:** Jiayuan Zhu, Xiaoya Li, Na Deng, Xinxin Peng, Zhoujin Tan

**Affiliations:** ^1^College of Pharmacy, Hunan University of Chinese Medicine, Changsha, China; ^2^College of Chinese Medicine, Hunan University of Chinese Medicine, Changsha, China; ^3^The First Affiliated Hospital of Hunan University of Chinese Medicine, Changsha, China

**Keywords:** adenine combined with *Folium sennae*, diarrhea with deficiency kidney-yang syndrome, gut mucosal microbiota, kidney, energy metabolism

## Abstract

The present study aims to study and analyze the characteristics of gut mucosal microbiota in diarrhea mice with deficiency kidney-yang syndrome. Ten male mice were randomly divided into the control group and the model group. Diarrhea mice model with deficiency kidney-yang syndrome was established by adenine combined with *Folium sennae*. The kidney structure was observed by hematoxylin-eosin (HE) staining. Serum Na^+^-K^+^-ATP-ase and Ca^2+^-Mg^2+^-ATP-ase were detected by enzyme-linked immunosorbent assay (ELISA). The characteristics of gut mucosal microbiota were analyzed by performing third-generation high-throughput sequencing. The results showed that the model mice exhibit obvious structural damage to the kidney. Serum Na^+^-K^+^-ATP-ase and Ca^2+^-Mg^2+^-ATP-ase levels showed a decreased trend in the model group. The diversity and community structure of the gut mucosal microbiota improved in the model group. Dominant bacteria like *Candidatus Arthromitus, Muribaculum*, and *Lactobacillus reuteri* varied significantly at different taxonomic levels. The characteristic bacteria like *Bacteroides, Erysipelatoclostridium, Anaerotignum, Akkermansia muciniphila, Clostridium cocleatum, Bacteroides vulgatus*, and *Bacteroides sartorii* were enriched in the model group. A correlation analysis described that *Erysipelatoclostridium* was positively correlated with Na^+^-K^+^-ATP-ase and Ca^2+^-Mg^2+^-ATP-ase levels, while *Anaerotignum* exhibited an opposite trend. Together, adenine combined with *Folium sennae* damaged the structure of the kidney, affected energy metabolism, and caused disorders of gut mucosal microbiota in mice. *Bacteroides, Erysipelatoclostridium*, and *Anaerotignum* showed significant inhibition or promotion effects on energy metabolism. Besides, *Akkermansia muciniphila, Clostridium cocleatum, Bacteroides vulgatus*, and *Bacteroides sartorii* might be the characteristic species of gut mucosal microbiota responsible for causing diarrhea with deficiency kidney-yang syndrome.

## Introduction

Diarrhea is defined as reduced stool consistency, increased water content, and the number of evacuations per day, which is highly associated with gut microbiota dysbiosis (Mendez et al., 2019; Xie et al., 2019; Shao et al., 2020; Li Y. X. et al., 2021). Currently, with the change in people's lifestyle and diet structure, the number of patients with diarrhea is increasing year by year (Huang et al., 2019). The earliest discussion of diarrhea in traditional Chinese medicine (TCM) was found in the “Yellow Emperor's Classic of Internal Medicine”. Ancient and modern medical practitioners mostly believed that the key internal organ of diarrhea was the spleen, which also involves the liver, kidney, and other internal organs. Due to the differences in etiology, pathogenesis, and clinical manifestations of patients, different types of diarrhea could be found in TCM, among which the deficiency kidney-yang syndrome was a common syndrome of diarrhea (Li Y. L. et al., 2021). As early as in the “Yellow Emperor's Classic of Internal Medicine”, the theory of “treating diarrhea from the kidney” was mentioned (Wang et al., 2016). TCM emphasized that the human body is a whole system and that the tissues of the internal organs are interrelated. The theory of “treating diarrhea from the kidney” explained that the spleen and kidney were physiologically related and pathologically connected. In the process of syndrome differentiation and treatment of diarrhea, the regulation of kidney functions should not be neglected (Chen et al., 2021). Hence, the development of diarrhea was closely related to the kidney.

Gut microbiota consists of a variety of microorganisms that reside in the gastrointestinal tract, and they are host-specific and evolve with the individual. The composition and diversity of this microbial community are susceptible to a variety of factors (such as diet, drugs, pathogens, and environmental factors), which in turn affect human and animal health (Wu et al., 2020). There is evidence that imbalances in the gut microbiota increase susceptibility to a wide range of pathogens and contribute to many diseases, including diarrhea, irritable bowel syndrome, allergies, cardiovascular disease, and obesity (Zhu et al., 2021). Besides, intestinal diseases may have multiple effects on the host, such as altering the composition of the gut microbiota (Meng et al., 2020; Zhou et al., 2020). Therefore, it is very meaningful to investigate the correlation between diarrhea with kidney-yang deficiency syndrome and changes in gut microbiota.

**Figure 1:**
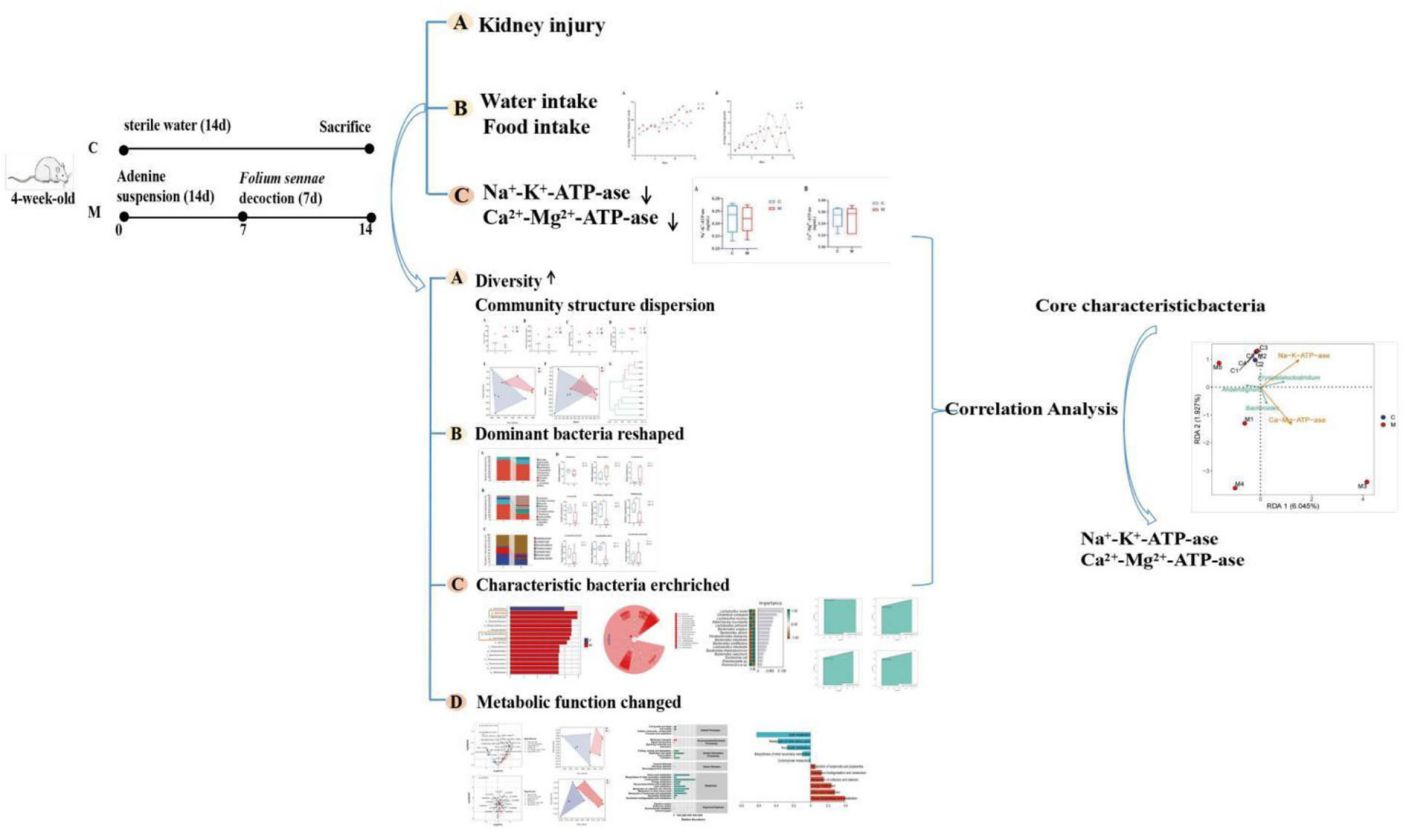
Experimental flow chart.

Adenine is a drug that is mainly used clinically in tumor radiation therapy, tumor chemotherapy, and psychotherapy (Su W. W. et al., 2020). Orally ingested adenine is rapidly metabolized to water-insoluble 2,8-dihydroxyadenine, which is deposited and crystallized in the microvilli and the apical domains of the epithelia in proximal renal tubules, causing renal tubule obstruction, leading to renal failure, and affecting the energy metabolism of renal tissue, thus resulting in the manifestation of kidney-yang deficiency (Jia and Jia, 2016; Sueyoshi et al., 2019). *Folium sennae is* a bitter-cold laxative commonly used in TCM, with the main laxative component being senna glycosides A and B, which can cause intestinal hyperfunction and lead to diarrhea (Guan et al., 2021). Our group has found significant diarrheal symptoms in mice after *Folium sennae* modeling and disturbances in the gut mucosal microbiota of the model mice (Zhang et al., 2020a). In addition, we compared the effects of adenine combined with *Folium sennae* at different doses and days on kidney and intestinal function in mice and found that adenine (50 mg/(kg·d), gavaged for 14 days) combined with *Folium sennae* (10 g/(kg·d), gavaged for 7 days) significantly caused impairment of the kidney and intestinal functions in mice (Li et al., 2022a). Subsequently, we have successfully constructed and validated a mouse model of diarrhea with deficiency kidney-yang syndrome using the same modeling method described above, thus confirming the reliability of the model (Li et al., 2022b). In this study, adenine combined with *Folium sennae* was used to construct a model of diarrhea with deficiency kidney-yang syndrome. By exploring the characteristics of gut mucosal microbiota and the correlation between differential bacteria and energy metabolism, this study provided a basis for exploring the treatment of diarrhea with kidney-yang deficiency syndrome from the perspective of gut mucosal microbiota. The specific process is shown in Figure 1.

## Materials and methods

### Animals

Ten 4-week-old Kunming male mice (weighing 18–22 g) were purchased from Slack Jingda Experimental Animal Co, Ltd. (Hunan, China, license number: SCXK [Xiang] 2016-0002). All mice were housed in the Animal Experiment Center of the Hunan University of Chinese Medicine with free access to food and drinking water (room temperature 23–25°C, relative humidity 50–70%, and 12 h of light/darkness). All animal experiments were performed according to the guidelines approved by the Institutional Animal Care and Use Committee of Hunan University of Chinese Medicine (license number: SYXK [Xiang] 2019-0009). Animal experiments were approved by the Animal Ethics and Welfare Committee of the Hunan University of Chinese Medicine (LLBH-202106120002). To exclude the effect of gender on gut microbiota, only male rats were used in this study (Wu et al., 2022).

### Medicine

Adenine (Changsha Yaer Biology Co., LTD, EZ2811A135) suspension preparation (Xiao et al., 2008): adenine was prepared in sterile water to a final concentration of 5 mg/mL in proportion to the concentration of the suspension, which is ready to use preparation for daily use. *Folium senna* (Anhui Puren Chinese Herbal Beverage Co., LTD, 2005302) decoction preparation (Xie et al., 2022): Appropriate amount of *Folium sennae* was placed in a decoction vessel, and then appropriate amount of water was added (over the *Folium sennae*) and soaked for 30 min. After 30 min, the water was poured off, and five times the amount of water was added to the vessel and boiled for 30 min. Then filtered dregs were added to an appropriate amount of water, and the boiling of the decoction was continued for 15 min. The decoction was mixed with the two decoctions and then boiled for 15 min. The decoction was further concentrated to form a decoction containing 1 g/mL of raw herbs and stored in a refrigerator at 4°C.

### Reagents

Na^+^-K^+^-ATP-ase enzyme-linked immunosorbent assay (ELISA) Kit (Jiangsu Jingmei Biotechnology Co., LTD, number: JM-11845M1) and Ca^2+^-Mg^2+^-ATP-ase ELISA Kit (Jiangsu Jingmei Biotechnology Co., LTD, number: JM-12156M1) were used in the experiments.

### Grouping and modeling of animals

After 3 days of acclimatization feeding, 10 male mice were randomly divided into the control (C) group and model (M) group, with five mice in each group. After the modeling method was improved in reference to the literature (Xiao et al., 2008, 2016), mice in the M group were gavaged with adenine suspension (50 mg/(kg·d), 0.4 mL per time) once a day for 14 consecutive days. From the 8th day of modeling, mice in the M group were gavaged with the *Folium sennae* decoction (10 g/(kg·d), 0.4 mL per time) once a day for 7 days. Mice in the C group were intragastrically gavaged with an equal volume of sterile water, once a day, for 14 days.

### Model evaluation criteria

According to the clinical manifestations of diarrhea with deficiency of kidney-yang syndrome (Spleen and Stomach Branch of China Association of Traditional Chinese Medicine, 2017), the diagnostic criteria of macroscopic symptoms in mice diarrhea with deficiency of kidney-yang syndrome were dilute feces, or incomplete pellets, cold extremities, curved and arched back, decreased appetite and body weight, and depression. On the basis of the manifestation of macroscopic signs, combined with histopathological sections of the kidney, a reliable basis for model evaluation was provided.

### General behavioral observations

During the experiment, mice were observed for their mental state, activity frequency, and fecal characteristics. The amount of water consumed and the amount of food ingested by the mice were tested and recorded daily (Li et al., 2022c).

### Pathological slides of kidney

Under aseptic conditions, the connective tissue of the kidney was removed on an ultra-clean bench, fixed in 4 % paraformaldehyde solution, dehydrated by gradient ethanol, made transparent by xylene, embedded in paraffin, sliced, stained with HE, and the histopathological changes of the kidney were observed under a light microscope.

### ELISA analysis

The blood sample for the ELISA was left to stand for 30 min at room temperature. After centrifugation at 3,000 r/min for 10 min, the serum was separated and the test samples were loaded into a sterilized centrifuge tube. The method for setting plate layout, adding samples, adding enzymes, incubation, washing plate, color, termination reaction, and machine detection was performed according to the instructions provided by the manufacturer of the ELISA kit.

### 16S rRNA gene high-throughput sequencing

After the experiment, the mice were sacrificed by cervical dislocation. Under aseptic conditions, the abdominal cavity of mice was opened and the small intestine was removed. The small intestine was cut open along the long axis, rinsed with saline, and dried with filter paper, and then the intestinal mucosa was scraped and collected on a sterilized coverslip (He et al., 2019). The samples were separately loaded into 1.5 mL sterilized centrifuge tubes, numbered, weighed, and then stored in a −80°C refrigerator for the detection of gut mucosal microbiota. The total genomic DNA of the samples was extracted from the intestinal mucosa samples using the bacterial DNA Kit (OMEGA, USA). The quantity and quality of the extracted DNA were determined by NanoDrop NC2000 spectrophotometer (Thermo Fisher Scientific, Waltham, MA, USA) and agarose gel electrophoresis. Forward primer 27F (5′-AGAGTTTGATCMTGGCTCAG-3′) and reverse primer 1492R (5′-GGACTACHVGGGTWTCTAAT-3′) were used for PCR amplification of bacterial 16S rRNA near the full-length gene. The 16S rRNA gene was amplified by polymerase chain reaction (PCR) using Q5 high-fidelity DNA polymerase (New England BioLabs, USA). PCR products were detected by 2% agarose gel electrophoresis and purified by the Axygen^®^AxyPrep DNA gel extraction kit. The recovered PCR amplification products were quantified by fluorescence intensity using the Quant-it PicoGreen dsDNA Assay Kit. According to the fluorescence quantitative results, the samples were mixed in proportion to the sequencing requirements of each sample (Yuan et al., 2020). Sequencing was completed by Paiseno Biological Co., LTD (Shanghai, China).

### Bioinformatics and statistical analysis

Gut mucosal microbiota was analyzed by high-throughput sequencing of 16S rRNA, and sequences with similarity higher than 97% were assigned to an OTU (Wang et al., 2018). Species accumulation curves were used to test the sequencing depth and evaluate the quality of sequence data. Chao1 and Observed_species indexes reflect the abundance of the community, and the larger the index, the higher the abundance of the community. Simpson and Shannon indexes reflect community diversity, and higher index values indicate higher community diversity. The beta diversity analysis examines the similarity of community structure among different samples. Three main methods, that is, principal coordinate analysis (PCoA), non-metric multidimensional scaling (NMDS), and clustering analysis, are used to naturally decompose the community data structure and rank the samples by ordination to observe the differences between samples (Bray and Curtis, 1957). LEfSe and random forest analysis detected groups that differ significantly in the abundance of gut mucosa and also identify potential biomarkers (Breiman, 2001; Edgar, 2013). The receiver operating characteristic curve (ROC) was plotted, and the area under the curve (AUC) was calculated to analyze the role of differential flora in predicting the disease. Redundant analysis (RDA) was used to investigate the association of biochemical indicators with gut mucosal microbiota (Zhang et al., 2021).

The SPSS 21.00 software was used for statistical analysis, and the data obtained from each group were expressed as mean ± standard deviation. If the data of the two groups were in line with normal distribution and homogeneity of variance, an independent sample *t-*test was used. If the data did not conform to a normal distribution and variance was uneven, Wilcoxon rank-sum test was used. *P* < 0.05 indicates a statistical difference, and *p* < 0.01 indicates a very strong statistical difference; otherwise, there was no statistical significance (Li X. Y. et al., 2021).

## Results

### Modeling induced behavioral changes in mice

**Figure 2:**
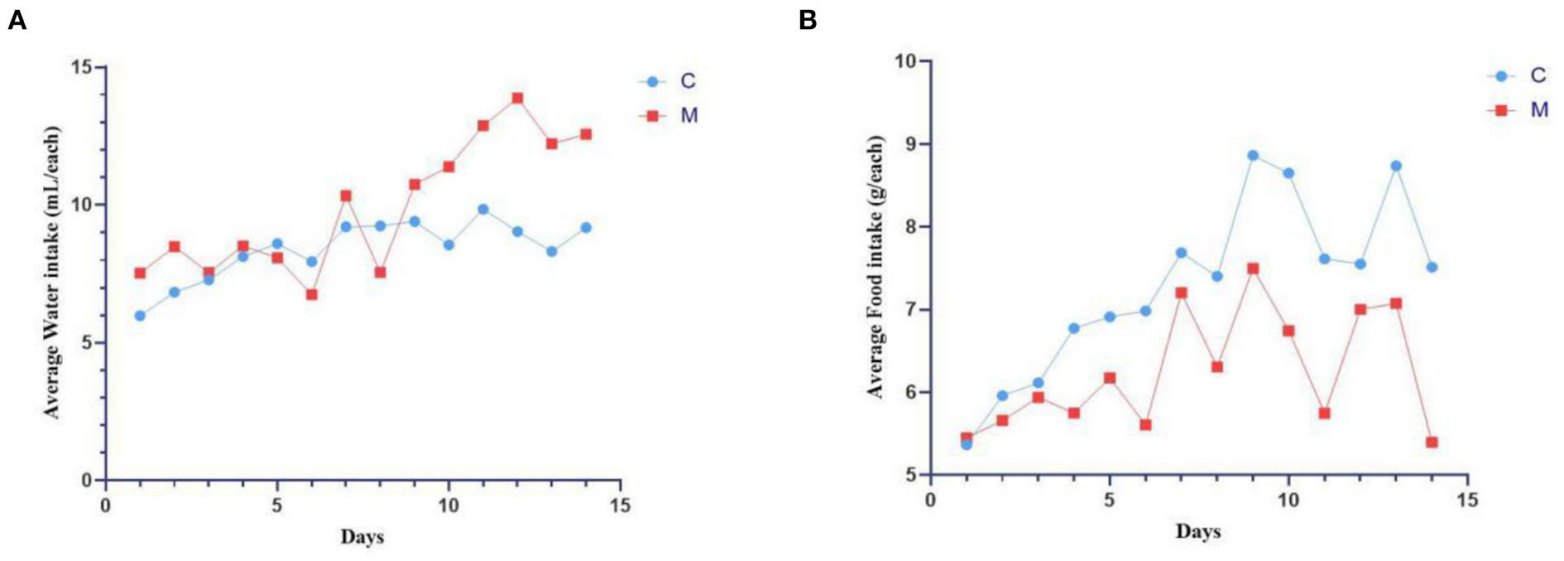
Modeling induced behavioral changes in mice. **(A)** Average water intake. **(B)** Average food intake. C, Control group (*n* = 5); M, Model group (*n* = 5). The values are expressed as mean ± standard deviation.

During the modeling period, mice in the C group had normal mental status and autonomic activity, with smooth and responsive fur. Mice in the M group were in poor mental status, with sparse and dull fur, wet bedding, and loose feces stuck to the bedding. On the 9th day of modeling (Figure 2A), the average daily water intake of the M group was much higher than the C group (*p* < 0.05). From the 4th day of modeling (Figure 2B), the average daily food intake of the M group was consistently lower than the C group (*p* > 0.05). The results suggested that modeling induced behavioral changes in mice.

### Modeling damaged the kidney structure in mice

**Figure 3:**
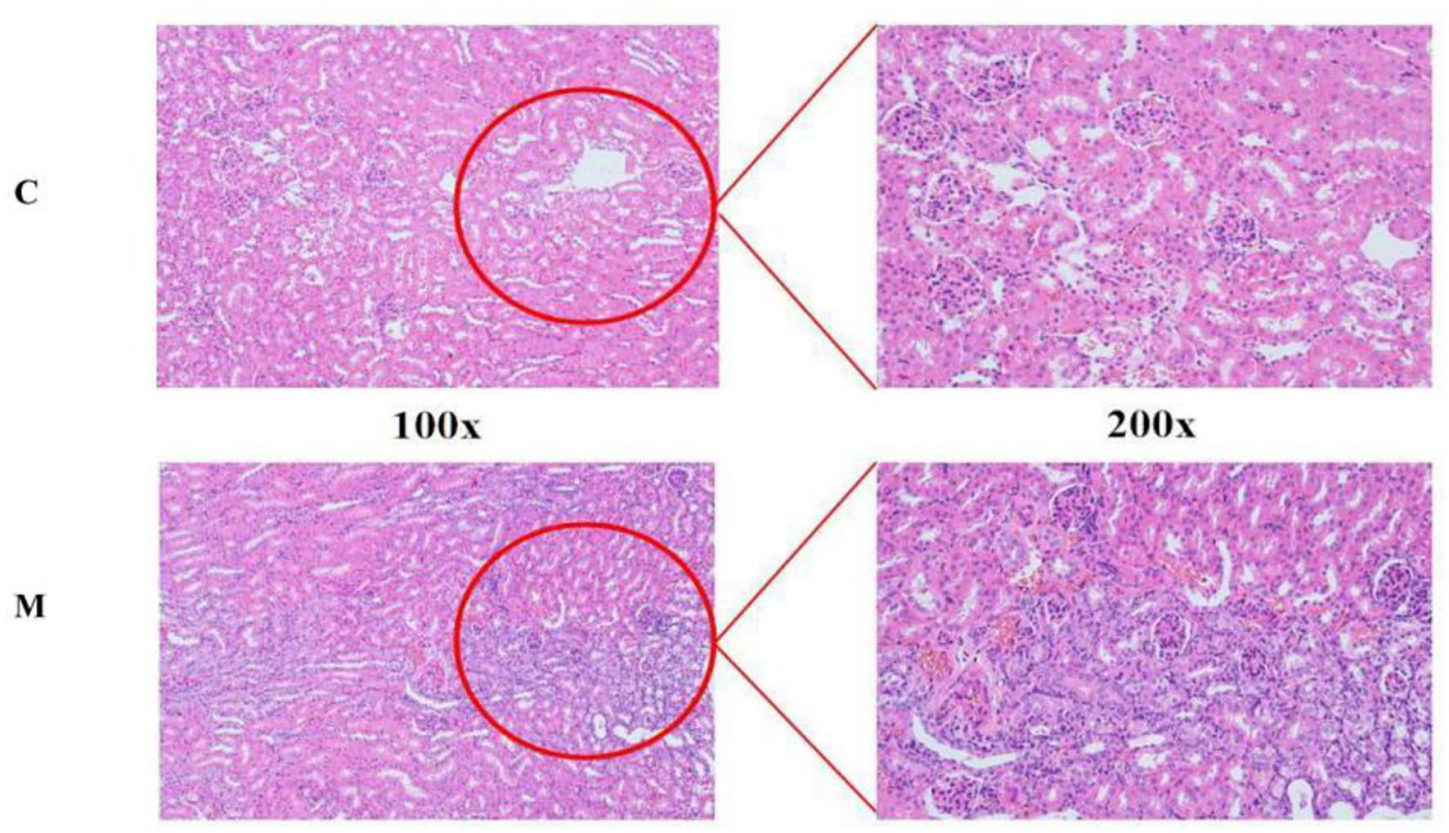
HE staining sections of kidney tissue. C, Control group; M, Model group.

The structural morphology of the kidney of mice in the C group showed no abnormal pathological manifestations. In the M group, glomerular thylakoid hyperplasia, interstitial edema, congestion, aggregation of inflammatory cells, different degrees of dilatation of renal tubules, lumen enlargement, tubular wall degeneration, and edema (Figure 3) were observed, indicating that modeling damaged the kidney structure of mice.

### Modeling affected energy metabolism in mice

**Figure 4:**
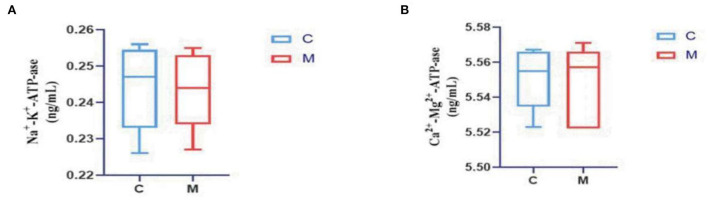
Modeling affected energy metabolism in mice. **(A)** Na^+^-K^+^-ATP-ase. **(B)** Ca^2+^-Mg^2+^-ATP-ase. C, Control group (*n* = 5); M, Model group (*n* = 5). The values are expressed as mean ± standard deviation.

The levels of Na^+^-K^+^-ATP-ase and Ca^2+^-Mg^2+^-ATP-ase were reduced in the M group when compared with the C group (*p* > 0.05; *p* > 0.05) (Figure 4), suggesting that adenine combined with *Folium sennae* affected the energy metabolism of mice to a certain extent.

### Sequencing data quality assessment and OTU count of gut mucosal microbiota in mice

**Figure 5:**
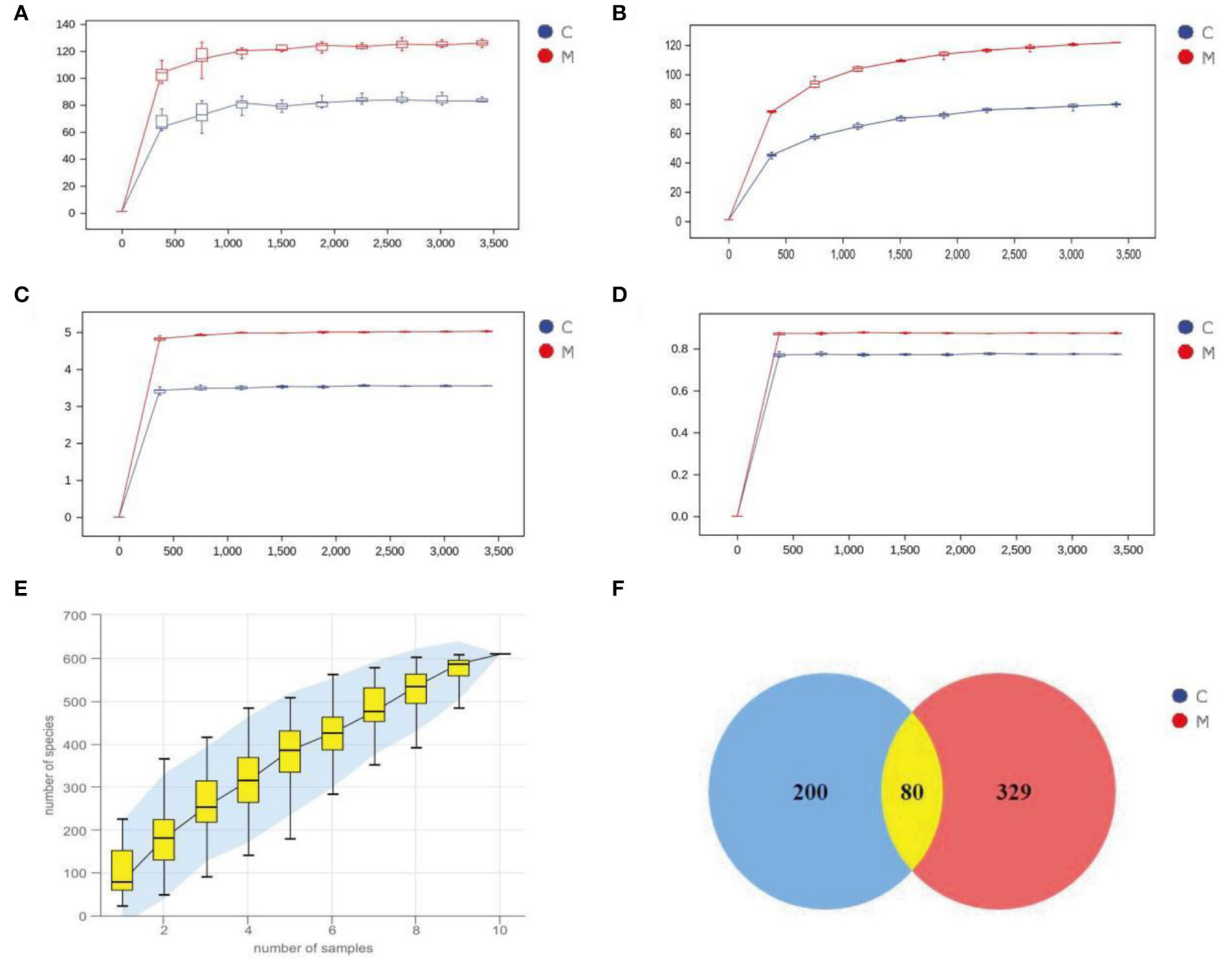
Sequencing data quality assessment and OTU count of gut mucosal microbiota. **(A)** Dilution curve of Chao1. **(B)** Dilution curve of Observed_species. **(C)** Dilution curve of Simspon. **(D)** Dilution curve of Shannon. **(E)** Species accumulation curves. **(F)** Venn diagram. C, Control group (*n* = 5); M, Model group (*n* = 5).

An inflection point occurred and then the curve flattened out with increasing sequencing depth to reach a plateau. The results indicated that the two sets of samples were sequenced at a sufficient and reasonable depth to cover most biological species and that the species richness of the samples tested was sufficient for subsequent studies (Figures 5A–D). Figure 5E depicts the species accumulation curve, which shows that with the increase in the sample size, the number of detected species increases significantly, and the curve becomes relatively steep. When the sample size increased to a certain level, further increase in the sample size does not detect new species and the curve tends to flatten out. These findings indicate that the sample size of this experiment was sufficient to reflect the richness of the community.

Venn diagram analyzes the unique or common OTUs between different sample groups, visually showing the similarity and uniqueness of the samples at the OTU level. The common OTUs in the C and M groups were 80. There were 200 OTUs unique to the C group and 329 OTUs unique to the M group. The total number of OTUs in the normal group was 280, and the total number of OTUs in the model group was 409 (Figure 5F), suggesting that modeling increased the number of species of the gut mucosal microbiota and taxonomic units in mice.

### Modeling affected the diversity and the microbiota structure in mice

**Figure 6:**
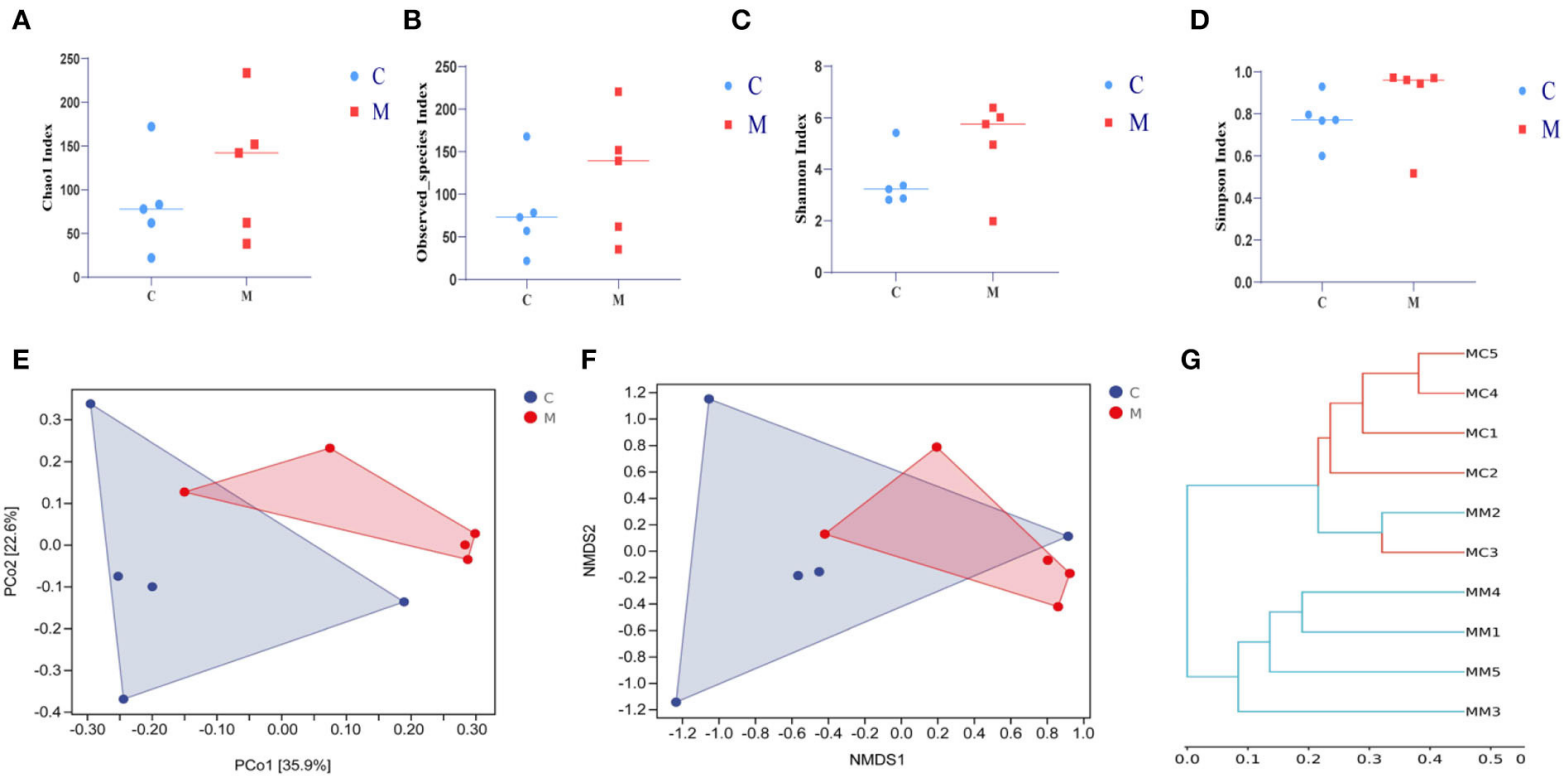
Effect of modeling on the diversity of the gut mucosal microbiota in mice. **(A)** Chao1 index. **(B)** Observed_species index. **(C)** Shannon index. **(D)** Simpson index. **(E)** PCoA analysis. **(F)** NMDS analysis. **(G)** Clustering analysis. C, Control group (*n* = 5); M, Model group (*n* = 5).

In order to comprehensively assess the alpha diversity of microbial communities, Chao1 and Observed_species indexes were used to determine the richness of species. Shannon and Simpson indexes were used to evaluate community diversity (Figures 6A–D). The Simpson index in the C group was slightly lower than that in the M group (*p* > 0.05), and the Chao1, Shannon, and Observed_species indexes in the C group were slightly higher in the M group (*p* > 0.05, *p* > 0.05, and *p* > 0.05, respectively). As could be seen from Figures 6E,F, the M samples were efficiently separated from the C samples and presented the phenomenon of grouping and aggregation. All these findings suggest that adenine combined with *Folium sennae* modeling altered the homogeneity of gut mucosal microbiota. From the clustering analysis (Figure 6G), it could be seen that the distance between the samples in the C group was relatively small. It reflected the small intra-group variation. Several samples in the M group, except for the M2 sample, could be well clustered into one group. In addition, M1 and M4 clustered more easily with the rest of the samples of the M group than with the C group, which reflected that the intra-group variation of the samples in the M group was larger than that in the C group, but they still could be well separated from the samples of the C group. Together, modeling affected the diversity and microbiota structure in mice.

### Modeling reshaped the dominant bacteria composition of the gut mucosal microbiota in mice

**Figure 7:**
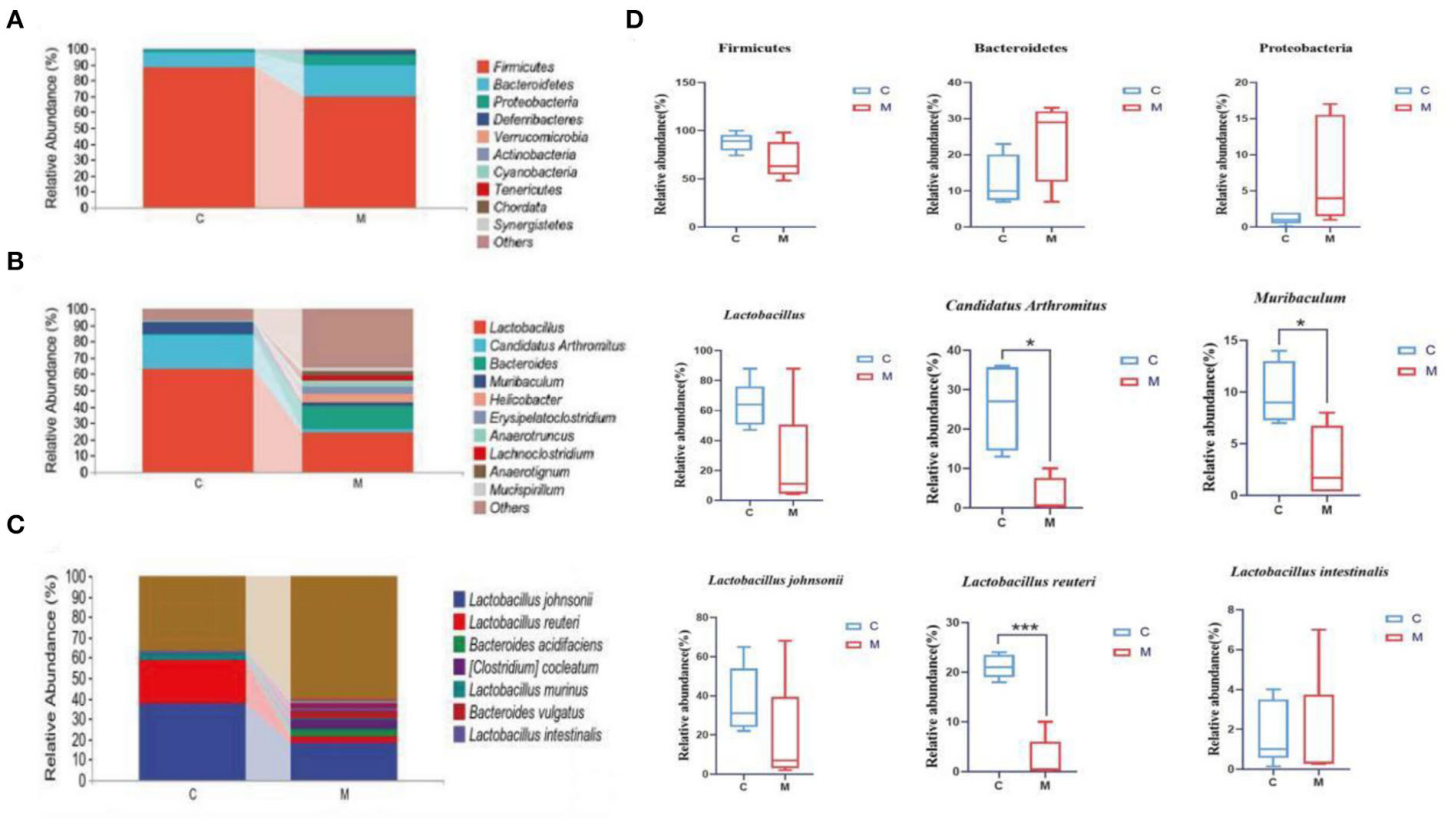
Effect of modeling on the relative abundance of gut mucosal microbiota in mice. **(A)** Relative abundance of gut mucosal microbiota at the phylum level. **(B)** Relative abundance of gut mucosal microbiota at the genus level. **(C)** Relative abundance of gut mucosal microbiota at the species level. **(D)** Phylum, genus, and species levels of dominant bacteria of gut mucosal microbiota in mice. C, Control group (*n* = 5); M, Model group (*n* = 5). The values are expressed as mean ± standard deviation. *p < 0.05, ***p < 0.01.

The horizontal coordinates of the bars indicated groups, and the vertical coordinates, respectively, indicated the relative abundance of gut mucosal microbiota at the phylum, genus, and species levels. We performed a taxonomic histological analysis in the C group and the M group and compared the differences at the phylum, genus, and species levels. Figure 7A indicates that the top three phyla were Firmicutes, Bacteroidetes, and Proteobacteria in both the C group and the M group (87.99, 10.06, and 1.09% in the C group, but 69.73, 19.65, and 7.31% in the M group, respectively).

*Lactobacillus, Candidatus Arthromitus*, and *Muribaculum* were the top three genera in the C group, and the dominant genera in the M group were *Lactobacillus, Bacteroides*, and *Helicobacter*. The M group had a lower proportion of *Lactobacillus, Candidatus Arthromitus*, and *Muribaculum* and a higher proportion of *Bacteroides* and *Helicobacter* than the C group (Figure 7B), indicating the changes in the composition of the dominant bacteria at the genus level.

Specific for species level analysis (Figure 7C) indicated that the abundance of *Lactobacillus johnsonii, Lactobacillus reuteri*, and *Lactobacillus murinus* in the C group was markedly higher than that in the M group. The dominant bacteria in the M group specifically were *Lactobacillus johnsonii, Bacteroides acidifaciens*, and *Bacteroides vulgatus*. The population of *Bacteroides vulgatus* and *Bacteroides sartorii* was significantly higher in the M group than in the C group, which indicated the changes in the composition of the dominant bacteria at the species level.

We further performed a statistical analysis of the bacteria with a relative abundance >1% in both the C group and the M group at the phylum, genus, and species levels (Figure 7D). Compared with the C group, the relative abundance of Bacteroidetes and Proteobacteria in the M group was increased (*p* > 0.05; *p* > 0.05), while that of Firmicutes decreased (*p* > 0.05). *Lactobacillus, Candidatus Arthromitus*, and *Muribaculum* in the M group decreased significantly (*p* > 0.05, *p* < 0.05, and *p* < 0.05, respectively). At the species level, *Lactobacillus johnsonii, Lactobacillus intestinalis*, and *Lactobacillus reuteri* in the M group decreased (*p* > 0.05, *p* > 0.05, and *p* < 0.01, bacteria). In summary, Bacteroidetes, Proteobacteria, Firmicutes, *Lactobacillus, Candidatus Arthromitus, Muribaculum, Lactobacillus johnsonii, Lactobacillus intestinalis*, and *Lactobacillus reuteri* might play important an role as the dominant bacteria in diarrhea with kidney-yang deficiency syndrome.

### Significant enrichment of core differential bacteria of gut mucosal microbiota in mice

**Figure 8:**
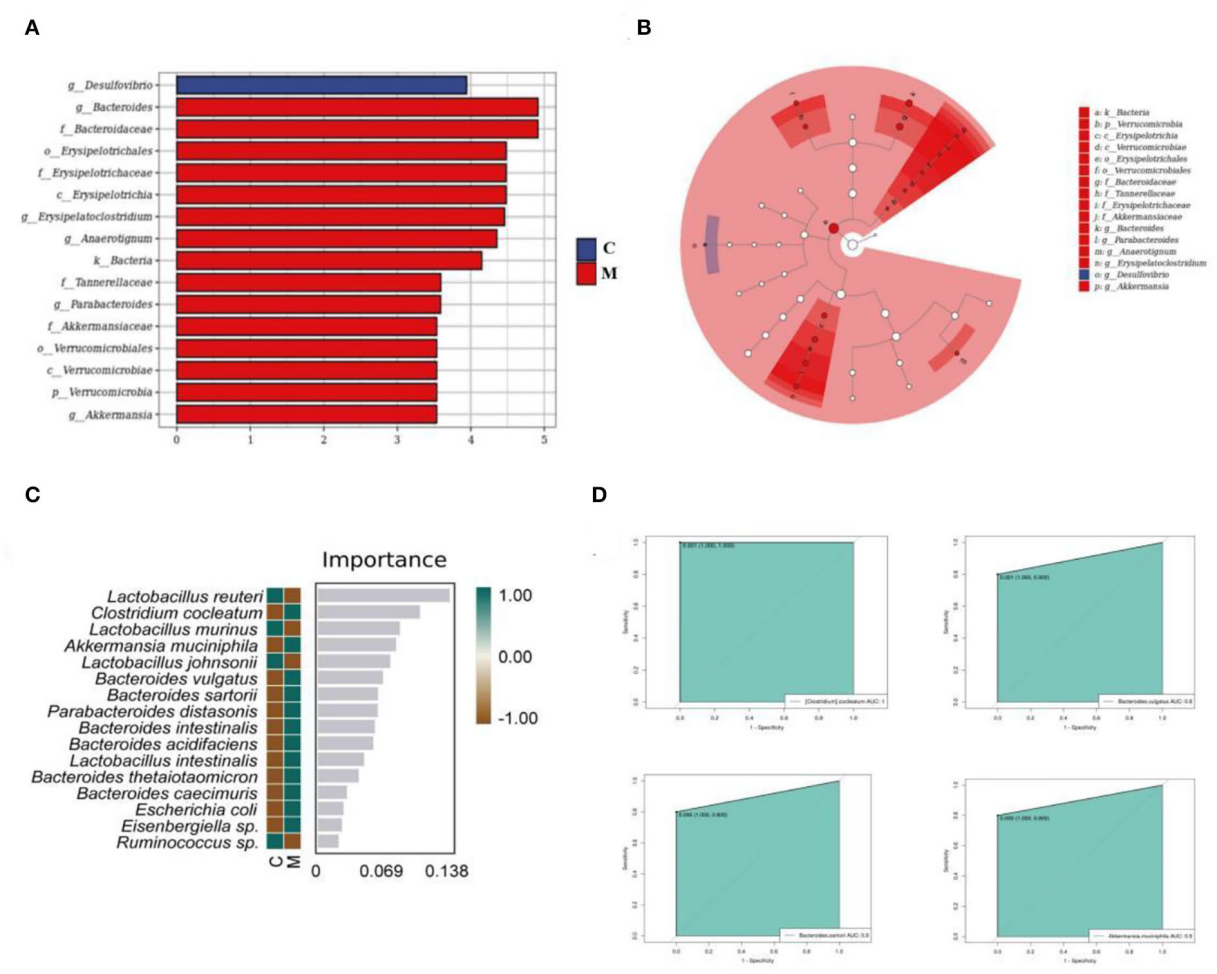
Core characteristic bacterial analysis of gut mucosal microbiota. **(A)** LDA diagram. **(B)** Cladogram diagram. **(C)** Random forest diagram of species level. **(D)** ROC curve of species. C, Control group (*n* = 5); M, Model group (*n* = 5).

The LEfSe method was used to directly search for key species that were statistically different between groups at all taxonomic levels. In the experiment, LDA = 4 was set as the cut-off point. The C group showed no significant enrichment of any bacterial taxon. The M group showed significant enrichment of eight bacterial taxa. Of these, *Bacteroides, Erysipelatoclostridium*, and *Anaerotignum* were involved at the genus level (Figures 8A,B). Then, we constructed a random forest diagnostic model to distinguish the C group from the M group by using 20 bacteria at the species levels (Figure 8C). The ROC results displayed (Figure 8D) that *Akkermansia muciniphila, Clostridium cocleatum, Bacteroides vulgatus*, and *Bacteroides sartorii* presented large AUC values, denoting that *Akkermansia muciniphila, Clostridium cocleatum, Bacteroides vulgatus*, and *Bacteroides sartorii* might be used as potential biomarkers at the species level of gut mucosal microbiota for the diagnosis of diarrhea with deficiency kidney-yang syndrome.

### Modeling altered the function of the gut mucosal microbiota in mice

**Figure 9:**
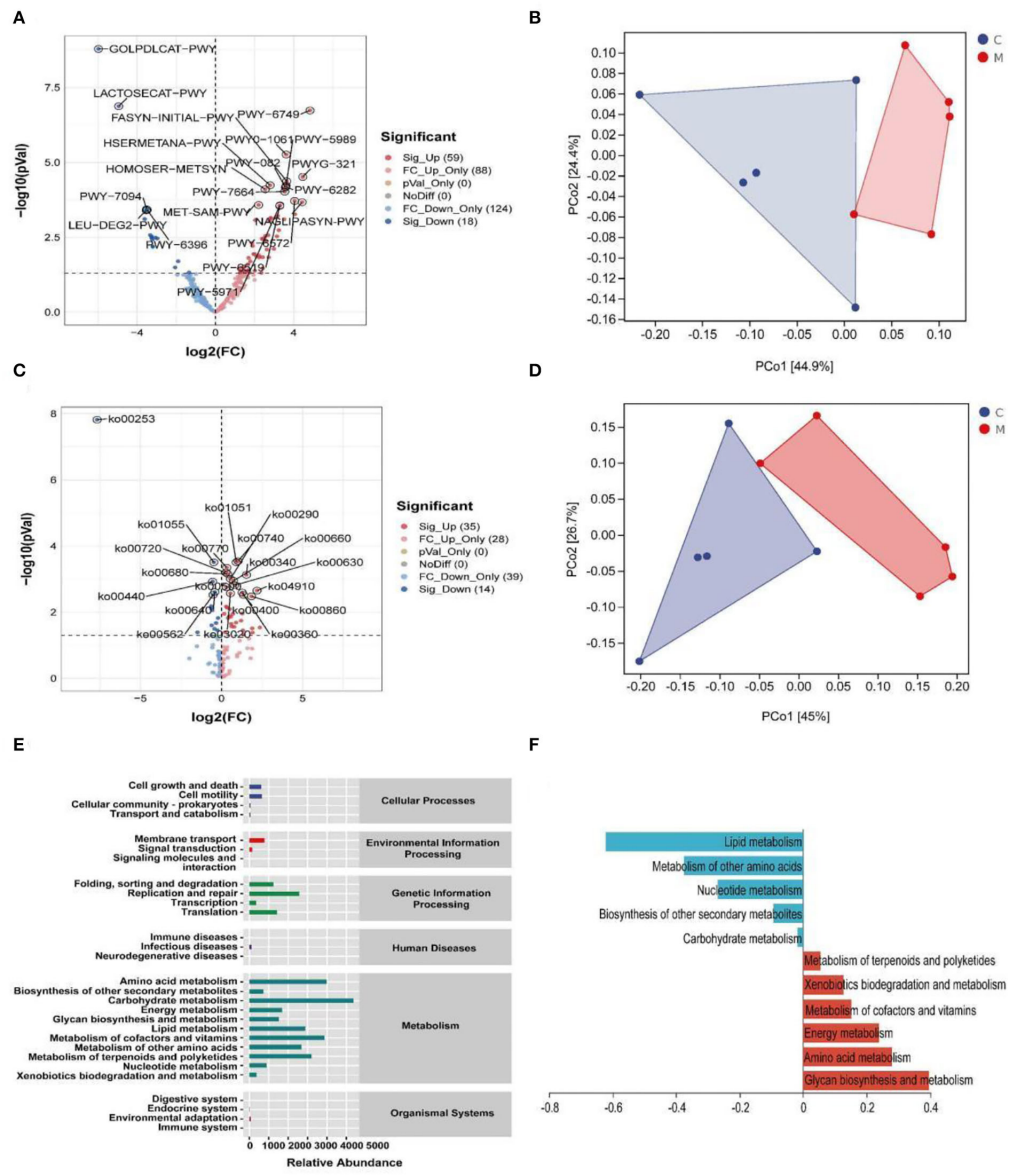
Functional analysis of gut mucosal microbiota. **(A)** Volcano map of MetaCyc pathway. **(B)** MetaCyc functional unit PCoA map. **(C)** KEGG pathway volcanoes. **(D)** PCoA diagram of KEGG functional units. **(E)** Predicted abundance of KEGG function. **(F)** Histogram of metabolic function in positive and negative coordinates. C, Control group (*n* = 5); M, Model group (*n* = 5).

A phylogenetic investigation of communities by reconstruction of unobserved states2 (Picrust2) software was applied to predict 396 EC enzyme labels of microbiota (including 59 upregulated ECs and 18 downregulated ECs, *p* < 0.05) and 170 KEGG homologous genes (including 35 upregulated KOs and 14 downregulated KOs, *p* < 0.05) (Figures 9A,C). The samples in the two databases showed significant separation (Figures 9B,D). Also, the gut mucosal microbiota function was generally divided into six categories, and the second level included 29 sub-functional categories, with the metabolic function accounting for a greater abundance. Among them, the gut mucosal microbiota of mice had a significant role in regulating carbohydrate metabolism, amino acid metabolism, and energy metabolism (Figure 9E).

### Modeling affected the interaction between differential bacteria of gut mucosal microbiota and energy metabolism in mice

**Figure 10:**
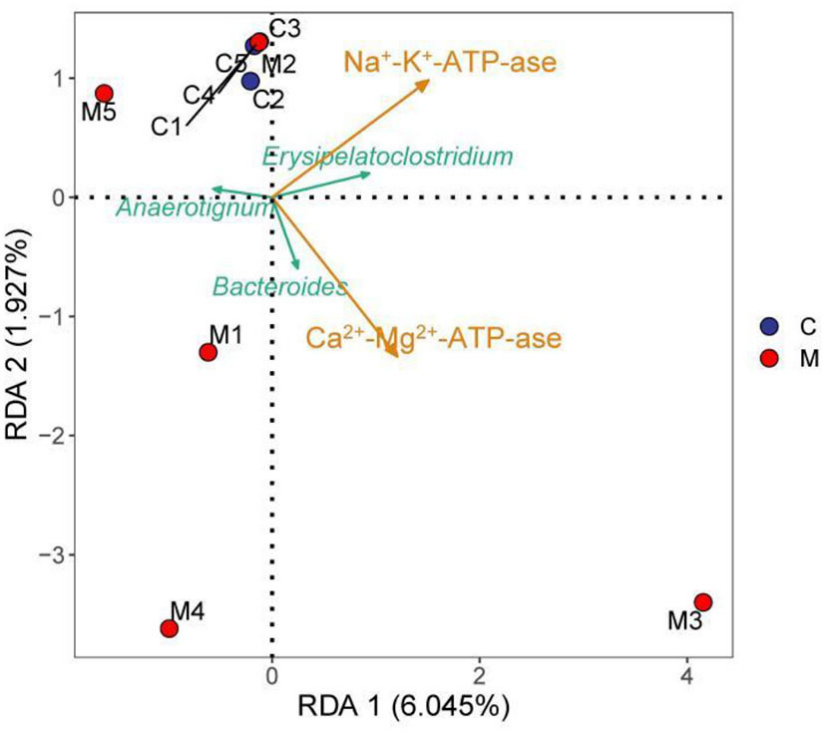
RDA analysis. C, Control group (*n* = 5); M, Model group (*n* = 5).

The RDA results pointed out that *Bacteroide*s was positively correlated with Ca^2+^-Mg^2+^-ATP-ase and negatively correlated with Na^+^-K^+^-ATP-ase. *Erysipelatoclostridium* was positively correlated with Na^+^-K^+^-ATP-ase and Ca^2+^-Mg^2+^-ATP-ase. *Anaerotignum* was negatively correlated with Na^+^-K^+^-ATP-ase and Ca^2+^-Mg^2+^-ATP-ase (Figure 10). These results indicated that there was a correlation between the populations of *Bacteroides, Erysipelatoclostridium*, and *Anaerotignum* and the energy metabolism after mice were modeled with adenine combined with *Folium sennae*.

## Discussion

Observation of the animal behavioral characteristics defining the syndrome of TCM in animal models is an important support for TCM research (Ren and Peng, 2020). Behavioral changes in the mice were recorded and assessed during the modeling process. The mice in the M group showed increased water intake, cool tail, arching back and lethargy, piling up, loose stools, decreased anal temperature and filthy perianal area, and feces stuck to the bedding. From the 4th day of modeling, the average daily water intake in the M group was much higher than that in the C group. The average daily food intake in the M group was persistently lower than that in the C group since the 4th day. In conjunction with the kidney histopathological sections, we also observed that the glomerulus and renal tubules of mice in the M group were obviously damaged. The renal interstitium was edematous, and inflammatory cells were aggregated. It suggested that adenine combined with *Folium senna* damaged the structure of the kidney in mice. Na^+^-K^+^-ATP-ase and Ca^2+^-Mg^2+^-ATP-ase are membrane-bound proteins located in the inner mitochondrial and cellular membranes, and they are important indicators of mitochondrial function and energy metabolism levels (Simão et al., 2011). It was demonstrated that the activity of Na^+^-K^+^-ATP-ase and Ca^2+^-Mg^2+^-ATP-ase in the rats with kidney-yang deficiency syndrome was reduced and the mitochondrial structure was impaired, leading to the reduction in energy metabolism (Qiu et al., 2019). In the present study, the activity of both enzymes was affected in the serum of mice, which proved that the animal model of diarrhea with deficiency kidney-yang syndrome was successfully constructed.

The relationship between diarrhea and gut mucosal dysbiosis is gradually being understood (Zhang et al., 2020b, 2021). Both adenine and *Folium senna* caused gut mucosal barrier damage and an imbalance of microbial communities (Blander et al., 2017; Huang et al., 2022). Combined with the experimental results, the richness of gut mucosal microbiota (Chao1 and Observed_species indexes) and the diversity (Simpson and Shannon indexes) were higher in the M group than in the C group. Beta diversity also confirmed that modeling caused impressive dispersion of community structure of gut mucosal microbiota in mice, suggesting that modeling influenced the richness, diversity, and structure of gut mucosal microbiota.

Patients with kidney injury had an imbalance in gut mucosal microbiota, with an upregulation in Firmicutes, Actinobacteria, and Proteobacteria, and downregulation in *Bifidobacteria* and *Lactobacilli* (Vaziri et al., 2013). Studies have confirmed that diarrhea rats with spleen deficiency induced significant changes in the abundance of Firmicutes and Proteobacteria, and upgraded several genera, such as *Clostridium, Bacteroides, Parabacteroides, Alloprevotella*, and *Helicobacter* (Shi et al., 2020). The preliminary experiments showed that the abundance of Actinobacteria, Proteobacteria, *Veillonococcus, Mycoplasma, Escherichia coli*, and *Enterococci* was elevated in diarrhea mice with deficiency kidney-yang syndrome, while *Bifid bacteria and Lactobacillus* were decreased (Li X. Y. et al., 2021). In our experiments, the taxonomic composition of gut mucosal microbiota in mice changed after the animal was modeled. Compared to the C group, *Lactobacillus, Candidatus Arthromitus*, and *Muribaculum* in the M group decreased significantly. Besides, we found that *Lactobacillus johnsonii, Lactobacillus intestinalis*, and *Lactobacillus reuteri* in the M group presented notable downregulation, while *Clostridium* showed pronounced upregulation. It could be seen that modeling significantly altered gut mucosal microbiota composition at the phylum, genus, and species levels. LEfSe analysis revealed at the genus level that *Erysipelatoclostridium,Bacteroides*, and *Anaerotignum* were markedly enriched in the M group as differentiated bacteria. *Erysipelatoclostridium* is a genus of pathogenic bacteria that cause a variety of serious infections in immunocompromised patients (Milosavljevic et al., 2021). Previous studies have confirmed that green tea leaf powder promoted fatty acid catabolism and reduced the abundance of *Erysipelatoclostridium*, and its abundance was negatively correlated with lipid metabolism (Wang et al., 2020). It was reported that *Anaerotignum* produced acetate, propionate, and butyrate to provide energy to the host (Choi et al., 2019). In our study, *Erysipelatoclostridium* presented positive correlations with Na^+^-K^+^-ATP-ase and Ca^2+^-Mg^2+^-ATP-ase. *Anaerotignum* was negatively correlated with Na^+^-K^+^-ATP-ase and Ca^2+^-Mg^2+^-ATP-ase. The reasons for these results were presumed to be related to the interactions between the differential bacteria and the specific mechanism of action, which still needs further investigation. *Bacteroides* is usually a “friendly” commensal in the gut, which transfers through the gut mucosa and multiplies in normal sterile tissues, thus leading to abdominal inflammation, diarrhea, and abscess in the abdominal cavity (Zafar and Saier, 2021). Studies confirmed that in a balanced state of gut microbiota, *Bacteroides* used complex dietary polysaccharides and host glycans to provide energy to the body and promote the breakdown and metabolism of adipose tissue (Ito et al., 2020; Yoshida et al., 2021). When the gut mucosal barrier was damaged, the gut microbiota was disturbed and bacteria appeared to translocate. *Bacteroides* facilitated pathogen growth by producing virulence factors and depriving the host of nutrients (Zafar and Saier, 2021). *Folium sennae* has been found to cause intestinal mucosal barrier damage and intestinal mucosal permeability changes (Su P. et al., 2020). In this experiment, *Bacteroides* showed a positive regulation with Ca^2+^-Mg^2+^-ATP-ase but negative regulation with Na^+^-K^+^-ATP-ase. Therefore, we hypothesized that adenine combined with *Folium senna* might cause the migration of *Bacteroides* and thus the facilitation or inhibition of host energy metabolism. In summary, the study of specific gut-related functional microorganisms will be an essential direction (Long et al., 2017, 2018a,b; He et al., 2018).

## Conclusion

Adenine combined with *Folium senna* caused behavioral changes in mice, significantly damaged the structure of the kidney, affected energy metabolism, and caused disorders of gut mucosal microbiota. Furthermore, the correlation between *Bacteroides, Erysipelatoclostridium*, and *Anaerotignum* and diarrhea with deficiency kidney-yang syndrome was revealed to have a more of a synergistic or competitive effect on energy metabolism.

## Data availability statement

The datasets presented in this study can be found in online repositories. The names of the repository/repositories and accession number(s) can be found below: https://www.ncbi.nlm.nih.gov/, PRJNA851244.

## Ethics statement

Animal experiments were approved by the Animal Ethics and Welfare Committee of Hunan University of Chinese Medicine (LLBH-202106120002).

## Author contributions

JZ and XL: conceptualization, methodology, and writing of the original draft. XL: data curation, methodology, and visualization. ND and XP: investigation and visualization. ZT: supervision, funding acquisition, reviewing, and editing. All authors contributed to manuscript revision and read and approved the submitted version.

## Funding

This study was supported by grants from the National Natural Science Foundation of China (No: 81874460) and the Natural Science Foundation of Hunan Province (2022JJ30440).

## Conflict of interest

The authors declare that the research was conducted in the absence of any commercial or financial relationships that could be construed as a potential conflict of interest.

## Publisher's note

All claims expressed in this article are solely those of the authors and do not necessarily represent those of their affiliated organizations, or those of the publisher, the editors and the reviewers. Any product that may be evaluated in this article, or claim that may be made by its manufacturer, is not guaranteed or endorsed by the publisher.
